# Serum Triglyceride Level: A Predictor of Complications and Outcomes in Acute Pancreatitis?

**DOI:** 10.1155/2016/8198047

**Published:** 2016-02-25

**Authors:** Hassan Tariq, Vinaya Gaduputi, Richard Peralta, Naeem Abbas, Suresh Kumar Nayudu, Phyo Thet, Tin Zaw, Shirley Hui, Sridhar Chilimuri

**Affiliations:** Department of Medicine, Bronx-Lebanon Hospital Center, 1650 Selwyn Avenue, Suite No. 10C, Bronx, NY 10457, USA

## Abstract

*Aim.* To study serum triglyceride level as a predictor of complications and outcomes in acute pancreatitis.* Methods.* In this retrospective observational study, 582 patients admitted with acute pancreatitis, who had serum triglyceride levels measured within the first 24 hours, were divided into two groups. The study group consisted of patients with a triglyceride level ≥2.26 mmol/L (group 2) and the control group consisted of triglyceride level of <2.26 mmol/L (group 1). We collected data for baseline demographics, laboratory values, incidence of complications (local and systemic), admission to the intensive care unit (ICU), ICU length of stay, length of total hospital stay, and death in the two groups.* Results.* A triglyceride level of ≥2.26 mmol/L was found to be an independent predictor of developing altered mental status (*p*: 0.004), pancreatic necrosis (*p*: 0.001), acute respiratory distress syndrome (*p*: 0001), systemic Inflammatory response syndrome (*p*: 0.001), acute kidney injury (*p*: 0.001), hospital length of stay (LOS) (*p*: 0.002), admission to intensive care unit (ICU) (*p*: 0.002), and ICU LOS (*p*: 0.003).* Conclusion.* A triglyceride level of ≥2.26 mmol/L on admission in acute pancreatitis is an independent predictor of developing local and systemic complications, hospital LOS, admission to ICU, and ICU LOS.

## 1. Introduction

Acute pancreatitis, an inflammatory disorder of the pancreas, is the most frequent cause of admission to hospital due to gastrointestinal disorders in the USA [1, 2]. With an annual incidence ranging from 4.9 to 35 per 100,000 population, approximately 15 to 25 percent of all patients with acute pancreatitis (AP) develop severe AP [3]. The mortality ranges from 3 percent in patients with interstitial edematous pancreatitis to 17 percent among patients with pancreatic necrosis [4, 5]. Between 1988 and 2003, mortality from acute pancreatitis decreased from 12 percent to 2 percent, according to a large epidemiologic study [3]. However, mortality rates remain much higher in subgroups of patients with severe disease. The ability to predict the severity of acute pancreatitis can help identify patients at increased risk for morbidity and mortality, therefore helping clinicians to make an early decision to triage these patients to intensive care units as well as selection of patients for specific interventions.

A multitude of predictive models have been developed to predict the severity of acute pancreatitis (AP) based upon clinical, laboratory, and radiological parameters [6]. Serum triglyceride (TG) concentrations above 11 mmol/L (1000 mg/dL) can precipitate attacks of acute pancreatitis [7]. Hypertriglyceridemia (HTG) accounts for 1 to 4 percent of cases of acute pancreatitis [8, 9]. On the other hand, HTG is commonly present at the early stage of non-HTG-induced AP and its clinical significance remains unclear [10].

The relationship between the elevated TG level and severity of non-HTG-induced AP is not well established. Some studies reported that an elevated triglyceride level in non-HTG-induced AP was accompanied by more severe disease [11, 12]. However, other studies did not show any significant relationship between an elevated TG level and the severity or prognosis of AP patients [13].

The impact of different levels of HTG on the severity and complications of AP has not been clearly defined. In this study, we aimed to analyze the influence of elevated triglyceride level in acute pancreatitis (AP) and its prognosis.

## 2. Methods

This is a retrospective single center observational study. The period of study was 6 years between October 1, 2008, and October 31, 2014. The study was performed according to the Declaration of Helsinki and was approved by the Institution Review Board (IRB) of Bronx Lebanon hospital center.

### 2.1. Patient Selection

The data was collected from the electronic medical records of patients and tabulated in Microsoft Excel® (Microsoft Corp, Redmond, WA, USA). Patients ≥18 years of age admitted to our hospital with the diagnosis of acute pancreatitis (AP), who had serum triglyceride levels measured within the first 24 hours of admission, were included in the study population. The diagnosis of AP was made when any two of the following three criteria were met: classic abdominal pain; elevation of amylase and/or lipase three times the upper limit of normal; and radiographic evidence of acute pancreatitis. The initial study population consisted of 686 patients. Patients with end stage renal disease, those with chronic kidney disease and those with missing information/data were excluded from the study. A total of 582 patients were finally included and divided into two study groups.

### 2.2. Group Division

The study group consisted of patients with a triglyceride level ≥2.26 mmol/L (≥200 mg/dL) (group 2) and the control group consisted of triglyceride level of <2.26 mmol/L (<200 mg/dL) (group 1). All the values were measured within the first 24 hours of presentation.

### 2.3. Data Collection

Baseline demographic data including age, gender, and ethnicity were collected for all patients in the study. We collected the data on patients' Body mass indices (BMI), vital signs on admission, complete blood count, complete metabolic panel, D-dimers, Liver function test, serum amylase, serum lipase, and serum triglyceride levels. We calculated the incidence of local and systemic complications including acute respiratory distress syndrome (ARDS), shock, pancreatic necrosis, and acute kidney injury (AKI), among the two study groups. We also collected the data on the antibiotic usage during hospitalization, need for surgical or interventional radiological intervention, admission to the intensive care unit (ICU), ICU length of stay (LOS), length of total hospital stay, and death in the two groups.

### 2.4. Definitions and Criteria

The classification of AP severity was based on the 2012 revision of the Atlanta Classification [14]. Severe AP was defined by the presence of persistent (≥48 h) organ failure and/or death. Moderately severe acute pancreatitis is characterized by the presence of transient (<48 h) organ failure or local or systematic complications in the absence of persistent organ failure. Mild AP is defined by the absence of organ failure and/or pancreatic necrosis.

Complications were defined as follows: shock as a systolic blood pressure of less than 90 mm of Hg, systemic inflammatory response syndrome (SIRS) defined by the presence of ≥2 of the following clinical findings: body temperature higher than 38°C or lower than 36°C, heart rate higher than 90/min, hyperventilation evidenced by respiratory rate higher than 20/min, or a PaCO_2_ of less than 4.26 kPa (32 mm of Hg), white blood cell count higher than 12,000 × 10^9^/L or lower than 4,000 × 10^9^/L, sepsis defined as SIRS associated with infection, severe sepsis as sepsis associated with organ dysfunction, hypoperfusion, or hypotension, and septic shock as sepsis with arterial hypotension despite adequate fluid resuscitation. Pancreatic necrosis is defined as diffuse or focal areas of nonviable pancreatic parenchyma >3 cm in size or >30% of the pancreas. Acute kidney injury (AKI) was defined as increase in the serum creatinine concentration of ≥26.5 *μ*mol/L (≥0.3 mg/dL) from baseline; a percentage increase in the serum creatinine concentration of ≥50 percent; or oliguria of <0.5 mL/kg per hour for more than six hours.

The Bedside Index for Severity in Acute Pancreatitis (BISAP) score that predicts clinical outcomes in patients with AP was calculated using the variables of BUN >8.92 mmol/L (>25 mg/dL), impaired mental status, ≥SIRS criteria, age > 60, and presence of pleural effusion with each variable assigned one point if present [15].

### 2.5. Statistical Methods

Statistical analysis was performed with IBM SPSS 20 (Statistical Packages for the Social Sciences). Results were reported as the means with standard deviation for most variables and 95% confidence intervals or percentages for some variables. For comparison of continuous variables between the two groups, we used the independent sample tests *t*-test. Dichotomous variables were compared by chi-square analysis using the Pearson test. Subsequently, we used a multivariate analysis of covariance (MANCOVA) model to determine whether a triglyceride of ≥2.26 mmol/L (≥200 mg/dL) was independently associated with various complications in AP. Variables with *p* value <0.05 in the univariate analysis were included in the multivariate model. The model was thus adjusted for relevant risk factors for AP severity, namely, advanced age (≥60 years), sex, body mass index ≥30 (kg/m^2^) (class I obesity), and diabetic status. Linear regression and multiple regression analysis were used to find statistical significant predictors of complications between groups. A two-tailed value of *p* < 0.05 was considered statistically significant.

## 3. Results

### 3.1. Comparison of General Information of Patients

There were 582 AP patients included in the study, out of which 482 had a triglyceride level of <2.26 mmol/L (group 1) and 100 had a triglyceride level of ≥2.26 mmol/L (group 2). This cohort included 256 men and 226 women. The male to female ratio in group 1 was lower as compared to group 2 (1.13 versus 2.12, *p*: 0.004). The mean BMI was similar in both the groups (28.7 ± 7.6 versus 27.25 ± 5.9, *p*: 0.086). There were more cases of acute biliary pancreatitis in group 1 and more cases of alcoholic pancreatitis in group 2. Baseline characteristics and etiology of pancreatitis in both the groups are shown in Table 1.

Patients with triglyceride level of ≥2.26 mmol/L (group 2) had a higher rate of acute kidney injury on admission. Other laboratory parameters on admission and peak creatinine recorded during the admission are tabulated in Table 2.

### 3.2. Incidence of Complications in the Two Groups

Patients in group 2 had a higher rate of altered mental status on presentation as compared to group 1 (13% versus 5.1%  *p*: 0.007). There was no significant difference between the two groups in the incidence of pleural effusion (12.6% versus 17%  *p*: 0.259) and shock (2.69% versus 5%  *p*: 0.213). The incidence of pancreatic necrosis (12% versus 3.11%  *p*: 0.001) as well as acute respiratory distress syndrome (7% versus 1.45%  *p*: 0.005) was higher in group 2 as compared to group 1. We observed in our study that the incidence of acute kidney injury during the hospitalization was significantly higher in patients with a triglyceride level of ≥2.26 mmol/L on admission (52% versus 34.85%  *p*: 0.001). Patients in group 2 had a significantly higher incidence of SIRS (31% versus 16.8%, *p*: 0.002) as compared to group 1.

The hospital length of stay (LOS) in days (7.3 ± 4.4 versus 10.9 ± 7.8  *p*: 0.002), incidence of admission to the intensive care unit (22.4% versus 37%, *p*: 0.003), and the ICU length of stay in days (1.1 ± 0.3 versus 2.2 ± 0.5, *p*: 0.003) were higher in group 2 as compared to group 1.

The rates of gastrointestinal bleeding need for surgical intervention, antibiotic use, and death between the two groups were not statistically different. Incidence of complications in the two study groups is tabulated in Table 3.

### 3.3. Multivariate Analysis

We performed multivariate analysis using multivariate analysis of covariance (MANCOVA) model adjusting for relevant risk factors associated with AP severity, namely, advanced age (≥60 years), sex, body mass index ≥30 (class I obesity), and diabetic status (Wilks Lambda: 0.001). The results are tabulated in Table 4.

Patients in group 2 were found to have a higher BISAP score on admission (*p*: 0.002) (Table 5). Similarly, in group 2 there was a higher incidence of SIRS on admission (31% versus 16.73 *p*: 0.011) (Table 6).

### 3.4. Linear Regression Analysis

A triglyceride level of ≥2.26 mmol/L (≥200 mg/dL) was found to be an independent predictor of developing altered mental status (*β*: 0.119, 95% CI 0.09–0.32, *p*: 0.004), pancreatic necrosis (*β*: 0.160, 95% CI 0.08–0.24, *p*: 0.001), ARDS (*β*: 0.137, 95% CI 0.11–0.18, *p*: 0001), SIRS (*β*: 0.136, 95% CI 0.09–0.5 *p*: 0.001), acute kidney injury (*β*: 0.145, 95% CI 0.08–0.7, *p*: 0.001), hospital length of stay (*β*: 0.127, 95% CI 0.11–0.14, *p*: 0.002), admission to ICU (*β*: 0.127, 95% CI 0.05–0.6, *p*: 0.002), and ICU LOS (*β*: 0.125, 95% CI 0.09–0.14, *p*: 0.003).

### 3.5. Multiple Regression Analysis

To validate our results from linear regression, a model of multiple linear regression analysis was done using altered mental status, pancreatic necrosis, ARDS, SIRS, acute kidney injury, hospital length of stay, admission to ICU and ICU LOS as independent variable, and a triglyceride level of ≥2.26 mmol/L (≥200 mg/dL) as dependent one, and we found an adjusted *R* square of 0.145, *p* ≤ 0.001, *F* = 4.402 *p* ≤ 0.001.

## 4. Discussion

Many predictive models have been developed to identify patients at increased risk for morbidity and mortality from acute pancreatitis. The ideal predictor would be a single marker that can be reliably and rapidly measured with cost-effectiveness while causing no discomfort to the patient [6]. Recent studies have suggested that triglyceride level ≥2.26 mmol/L in patients with acute pancreatitis increases the incidence of complications compared to normal triglyceride levels [16]. In our study, we aimed to study the serum triglyceride level as a marker that can predict the development of complications and the need for admission to ICU among patients admitted with acute pancreatitis. Although alcohol use is associated with elevated triglyceride levels [10], we did not find an increased percentage of individuals with triglyceride ≥2.26 mmol/L (≥200 mg/dL) among individuals with alcohol pancreatitis as compared to individuals with biliary pancreatitis (17.8% versus 13%  *p*: 0.24).

It is generally believed that a serum triglyceride (TG) level of more than 11.3 mmol/L (1000 mg/dL) is needed to precipitate AP, the reduction of which to levels well below 11.3 mmol/L is often preventative [7]. Animal studies showed that HTG intensifies the course of both edematous and necrotizing pancreatitis [11]. During the occurrence of AP, due to the body's stress response, serum catecholamine, and glucagon levels as well as lipase activity are increased, leading to accelerated break down of fat tissue with subsequent release of TG and increase in serum lipid concentrations [10].

We observed that group 1 had more patients with biliary pancreatitis (29% versus 21%, *p*: 0.001), which was likely due to the presence of more females (47% versus 32%) in this group consistent with the epidemiological studies that showed gallstones are more common in females [17–19]. Group 2 consisted of more male patients (68% versus 53% in group 1) and more cases of alcoholic pancreatitis (34% versus 32% in group 1, *p*: 0.001) were seen likely because males are more likely to abuse alcohol [20]. The prevalence of diabetes mellitus (DM) (45% versus 29%, *p*: 0.001) and elevated hemoglobin A1c (0.088 ± 0.028 versus 0.073 ± 0.025, *p*: 0.001) was higher in the study group, which may represent DM as a risk factor for worse outcomes in patients with AP.

Patients with a triglyceride level of more than ≥2.26 mmol/L had a lower level of serum sodium (133.5 ± 6.2 versus 135.8 ± 4.3, *p*: 0.001) and lipase levels (12.2 ± 1.70 versus 17.3 ± 1.06, *p*: 0.017). Elevated triglyceride levels can alter routine measurements of sodium and amylase. The excess triglyceride in a serum sample can displace water containing sodium and cause pseudohyponatremia [21]. HTG levels >5.65 mmol/L may cause a falsely normal amylase level, likely from HTG interference of the calorimetric reading. Serial dilutions of the serum amylase sample can reduce the triglyceride interference [22].

Hypertriglyceridemia was found to be an independent risk factor for development of acute kidney injury in patients with AP and development of AKI in acute pancreatitis is associated with a higher mortality [23]. We excluded patients with chronic kidney disease and end stage renal disease in our study population to validate these results. Patients with a triglyceride level of ≥2.26 mmol/L had higher creatinine levels on admission (141.44 ± 106.1 versus 77.8 ± 61.9, *p*: 0.001), a higher value of maximum creatinine during the admission (167.9 ± 61.9 versus 97.24 ± 70.7, *p*: 0.001) and higher incidence of acute kidney injury (52% versus 34.85%, *p*: 0.001). Pancreatic lipase hydrolyzes excess TG in serum resulting in the accumulation of free fatty acids (FFAs), which are toxic to organ function and TG depositing around kidney tubules is hydrolyzed by pancreatic lipase with production of high levels of toxic FFAs around the renal cells, which may directly impair renal function. The levels of pancreatic enzymes are much higher in glomerulus because of concentration and aggravate the damage of renal function [23].

Early phase of acute pancreatitis is associated with coagulation abnormalities and D-dimer can be used as a clinical parameter that has been shown to predict the severity of acute pancreatitis [24]. A higher D-dimer level was present in patients with triglyceride level of ≥2.26 mmol/L (3942.72 ± 1379.95 versus 1365.16 ± 479.15, *p*: 0.03) consistent with more severe disease and higher rates of complications. Although the mechanism underlying the elevated d-dimer levels is complicated, severe coagulative disorder characterized by the diffuse formation of intravascular microthrombi and activation of fibrinolysis could be the predominant cause of this phenomenon [25].

Approximately 85 percent of patients with acute pancreatitis have acute interstitial edematous pancreatitis characterized by an enlargement of the pancreas due to inflammatory edema. Approximately 15 percent of patients have necrotizing pancreatitis with necrosis of the pancreatic parenchyma, the peripancreatic tissue, or both [26]. Our study found that a triglyceride level of ≥2.26 mmol/L was found to be an independent predictor of developing pancreatic necrosis. The occurrence of pancreatic infection is a leading cause of morbidity and mortality in acute necrotizing pancreatitis. Approximately one-third of patients with pancreatic necrosis develop infected necrosis [26].

According to the revised Atlanta classification of acute pancreatitis, a systemic complication of acute pancreatitis is defined as an exacerbation of an underlying comorbidity. In the Atlanta classification, organ failure is a distinct entity separate from a systemic complication [14]. Pancreatic inflammation results in the activation of a cytokine cascade that manifests clinically as a systemic inflammatory response syndrome (SIRS). Patients with persistent SIRS are at risk for failure of one or more organs. Organ failure (acute respiratory failure, shock, and renal failure) may be transient, resolving within 48 hours in patients with moderately severe pancreatitis, or persistent for >48 hours in patients with severe acute pancreatitis [14]. Our study showed that acute respiratory distress syndrome (7% versus 1.45%  *p*: 0.005) was higher in patients with high triglyceride level on admission. Our study also showed that a higher triglyceride level on admission was a predictor that the patient will have a longer hospital stay and is more likely to get admitted to ICU and have a higher length of stay in the hospital.

Recent studies have hypothesized that obesity is associated with worse outcomes in acute pancreatitis due to the release of excessive amounts of fatty acids from lipolysis of fat by pancreatic lipases [10, 27]. In our study, the BMI of both the groups were similar (28.7 ± 7.6 versus 27.25 ± 5.9, *p*: 0.086) and we were unable to validate obesity as an independent risk factor associated with worsening of acute pancreatitis. We propose that the increased lipolysis and release of free fatty acids may be secondary to genetic polymorphisms such as the mutations in the lipoprotein lipase gene [28]. Such genetic polymorphisms may place these patients at a higher risk of developing hypertriglyceridemia during acute pancreatitis that in turn leads to direct tissue injury due to mitochondrial damage and upregulation of the inflammatory cascade predisposing to multi-organ failure [10, 27]. The rapid increase in the free fatty acids has various effects. They damage platelets and vascular endothelium in microcirculation and are associated with an increase in viscosity leading to tissue ischemia and damage of pancreatic acinar cells. Hence, a vicious cycle begins in which acute pancreatitis causes increased lipolysis in genetically predisposed individuals which further damages the pancreas and worsens the severity [10, 27].

A few recent reports have attempted to study the effect of triglyceride level on outcomes of pancreatitis and its complications [10, 16, 23, 27]. Our study has several strengths as compared to previous studies. We included all patients with acute pancreatitis irrespective of the etiologies; hence, the results are more widely applicable. Our sample size was larger as compared to some previous studies. We described individual complications as compared to organ failure as a single entity hence providing a more detailed analysis of the complications that are associated with a higher triglyceride level in acute pancreatitis. Due to the same reason, we were also able to validate the results of previous studies that showed that a higher triglyceride level is associated with increased incidence of AKI [23] and pancreatic necrosis [11, 16].

In summary, a TG ≥2.26 mmol/L on admission in acute pancreatitis is an independent predictor of developing local and systemic complications (organ failure), hospital length of stay, admission to ICU, and the ICU LOS. High plasma TG level may be one of the independent risk predictors of severe AP. However, our study had limitations due to its retrospective design and further research is needed to validate TG as a single predictor of severity.

## Figures and Tables

**Table 1:** Population baseline characteristics.

Characteristic	Triglyceride <2.26 mmol/L (<200 mg/dL) (*N*: 482)	Triglyceride ≥2.26 mmol/L (≥200 mg/dL) (*N*: 100)	*p* value
Age, years^*∗*^	49 ± 14	50.5 ± 13	0.390
Sex, number (%)			0.004
Male	256 (53)	68 (68)	
Female	226 (47)	32 (32)	
BMI kg/m^2^^*∗*^	28.7 ± 7.6	27.25 ± 5.9	0.086
Pancreatitis etiology, number (%)			
Biliary	140 (29)	21 (21)	0.001
Alcoholism	156 (32)	34 (34)	0.001
Hypertriglyceridemia	0 (0)	18 (18)	0.001
Post-ERCP	5 (1)	1 (1)	0.001
Unknown + other^*β*^	181 (37)	26 (26)	0.001
Comorbidities, number (%)^‡^			
Hypertension	298 (61)	69 (69)	0.097
Diabetes mellitus	139 (29)	45 (45)	0.001
CHF	25 (5)	8 (8)	0.188
Cirrhosis	22 (4.5)	3 (3)	0.352
HIV	67 (14)	13 (13)	0.481
Smoker	205 (42)	42 (42)	0.503
Alcoholism	267 (55)	59 (59)	0.221

^*∗*^Plus-minus are means with ± SD.

^‡^Percentage may not add up to 100 because some patients had more than one disorder or risk factor.

Β: cases of Pancreatitis in which a cause could not be established or were caused by other etiologies such as hypercalcemia, drugs, trauma, pancreatic divisum, or autoimmune pancreatitis.

**Table 2:** Laboratory test values on admission.

	Triglyceride <2.26 mmol/L (<200 mg/dL) (*N*: 482)	Triglyceride ≥2.26 mmol/L (≥200 mg/dL) (*N*: 100)	*p* value
Hematocrit	0.39 ± 0.058	0.40 ± 0.062	0.099
WBC (×10^9^/L)	10.1 ± 4.9	10.9 ± 47	0.097
Platelets (×10^9^/L)	229.6 ± 93.2	218.7 ± 95.6	0.291
Sodium (mmol/L)	135.8 ± 4.3	133.5 ± 6.2	0.001
Potassium (mmol/L)	4.1 ± 0.6	4.2 ± 0.7	0.013
Chloride (mmol/L)	102.3 ± 6.8	100.6 ± 7.2	0.021
Bicarbonate (mmol/L)	24.3 ± 4.3	22.1 ± 5.5	0.001
BUN (mmol/L)	5.21 ± 4.17	8.67 ± 4.60	0.004
Creatinine (*μ*mol/L) on admission	77.8 ± 61.9	141.44 ± 106.1	0.001
Maximum creatinine (*μ*mol/L)	97.24 ± 70.7	167.9 ± 61.9	0.001
Calcium (mmol/L)	2.35 ± 0.3	2.35 ± 0.2	0.663
AST (*μ*kat/L)	2.22 ± 0.66	2.20 ± 0.37	0.965
ALT (*μ*kat/L)	2.00 ± 0.64	1.73 ± 0.35	0.482
Bilirubin (*μ*mol/L)	22.23 ± 10.26	29.08 ± 6.84	0.105
Protein (g/L)	74 ± 8	75 ± 12	0.241
Albumin (g/L)	41.4 ± 6	40.1 ± 7	0.083
INR	1.1 ± 0.3	1.1 ± 0.3	0.664
D-dimer (nmol/L)	1365.16 ± 479.15	3942.72 ± 1379.95	0.037
HbA1c	0.073 ± 0.025	0.088 ± 0.028	0.001
Amylase (*μ*kat/L)	8.53 ± 0.65	7.16 ± 0.92	0.346
Lipase (*μ*kat/L)	17.3 ± 1.06	12.2 ± 1.70	0.017

**Table 3:** Incidence of complications in the two study groups.

Complication	Triglyceride <2.26 mmol/L (<200 mg/dL) (*N*: 482)	Triglyceride ≥2.26 mmol/L (≥200 mg/dL) (*N*: 100)	*p* value
Alteration in mental status	25 (5.1)	13 (13)	0.007
SIRS	81 (16.8)	31 (31)	0.002
Pleural effusion	61 (12.6)	17 (17)	0.259
Shock	13 (2.69)	5 (5)	0.213
Pancreatic necrosis	15 (3.11)	12 (12)	0.001
ARDS	7 (1.45)	7 (7)	0.005
AKI	168 (34.85)	52 (52)	0.001
LOS (days)^*∗*^	7.3 ± 4.4	10.9 ± 7.8	0.002
ICU LOS (days)^*∗*^	1.1 ± 0.3	2.2 ± 0.5	0.003
Admission to ICU	108 (22.4)	37 (37)	0.003
GI bleed	9 (1.86)	2 (2)	0.59
IR or surgery	63 (13)	14 (14)	0.452
Antibiotic use	133 (27.5)	32 (32)	0.217
Death	11 (2.28)	5 (5)	0.169

^*∗*^Plus-minus are means with ± SD.

**Table 4:** Multivariate analysis showing association of triglyceride level with complications after adjusting for advanced age, sex, obesity, and diabetic status.

Complication	Triglyceride <2.26 mmol/L (<200 mg/dL) (*N*: 482)	Triglyceride ≥2.26 mmol/L (≥200 mg/dL) (*N*: 100)	*p* value
Alteration in mental status	25 (5.1)	13 (13)	0.001
SIRS	81 (16.8)	31 (31)	0.047
Pancreatic necrosis	15 (3.11)	12 (12)	0.002
ARDS	7 (1.45)	7 (7)	0.002
AKI	168 (34.85)	52 (52)	0.001
LOS (days)^*∗*^	7.3 ± 4.4	10.9 ± 7.8	0.016
ICU LOS (days)^*∗*^	1.1 ± 0.3	2.2 ± 0.5	0.009
Admission to ICU	108 (22.4)	37 (37)	0.002

^*∗*^Plus-minus are means with ± SD.

**Table 5:** Incidence of the Bedside Index for Severity in Acute Pancreatitis (BISAP) score in the two groups.

	Number of BISAP score variables present in both groups.						Total
	0	1	2	3	4	5	
Triglyceride <2.26 mmol/L (<200 mg/dL) (*N*: 482)	260 (53.9)	150 (31.1)	53 (10.9)	13 (2.6)	4 (0.82)	2 (0.041)	482
Triglyceride ≥2.26 mmol/L (≥200 mg/dL) (*N*: 100)	35 (35)	37 (37)	16 (16)	7 (7)	4 (4)	1 (1)	100
Total	295	187	69	20	8	3	582

**Table 6:** Incidence of systemic inflammatory response syndrome (SIRS) in the two groups.

	SIRS variables present in both groups.					Total
	0	1	2	3	4	
Triglyceride <2.26 mmol/L (<200 mg/dL) (*N*: 482)	212 (43.9)	189 (39.2)	62 (12.8)	16 (3.31)	3 (0.62)	482
Triglyceride ≥2.26 mmol/L (≥200 mg/dL) (*N*: 100)	37 (37)	32 (32)	20 (20)	9 (9)	2 (2)	100
Total	249	221	82	25	5	582

## Core Tip

Many predictive models have been developed to identify patients at increased risk for morbidity and mortality from acute pancreatitis. In this retrospective study, we aimed to study the serum triglyceride level within 24 hours of admission as a simple marker that can predict the development of complications (local and systemic) and the need for admission to ICU among patients admitted with acute pancreatitis. The ability to predict the severity of acute pancreatitis can help identify patients at increased risk for morbidity and mortality, therefore helping clinicians to make an early decision to triage these patients to intensive care units as well as selection of patients for specific interventions.

## Data Sharing Statement

Technical appendix, statistical code, and dataset are available from the corresponding author (Hassan Tariq) at Htariq@bronxleb.org. Informed consent was not obtained but the presented data are anonymized and risk of identification is low. No additional data are available.

## Biostatistics Statement

Richard Peralta M.D. and Hassan Tariq M.D. from Bronx Lebanon Hospital Center reviewed the statistical methods of this study.
